# Subclinical Atrial Fibrillation and Risk of Stroke: Past, Present and Future

**DOI:** 10.3390/medicina55100611

**Published:** 2019-09-20

**Authors:** Ahmed AlTurki, Mariam Marafi, Vincenzo Russo, Riccardo Proietti, Vidal Essebag

**Affiliations:** 1 Division of Cardiology, McGill University Health Center, Montreal, QC H3G1A4, Canada; 2 Department of Neurology and Neurosurgery, Montreal Neurological Institute, Montreal, QC H3A2B4, Canada; 3 Depatment of Medical Translational Sciences, University of Campania “Luigi Vanvitelli”—Monaldi Hospital, 80131 Naples, Italy; 4 Department of Cardiac, Thoracic, and Vascular Sciences, University of Padua, 35121 Padua, Italy; 5 Hôspital Sacré-Coeur de Montréal, Montreal, QC H4J1C5, Canada

**Keywords:** subclinical atrial fibrillation, atrial high rate episodes, stroke

## Abstract

Subclinical atrial fibrillation (SCAF) describes asymptomatic episodes of atrial fibrillation (AF) that are detected by cardiac implantable electronic devices (CIED). The increased utilization of CIEDs renders our understanding of SCAF important to clinical practice. Furthermore, 20% of AF present initially as a stroke event and prolonged cardiac monitoring of stroke patients is likely to uncover a significant prevalence of SCAF. New evidence has shown that implanting cardiac monitors into patients with no history of atrial fibrillation but with risk factors for stroke will yield an incidence of SCAF approaching 30–40% at around three years. Atrial high rate episodes lasting longer than five minutes are likely to represent SCAF. SCAF has been associated with an increased risk of stroke that is particularly significant when episodes of SCAF are greater than 23 h in duration. Longer episodes of SCAF are incrementally more likely to progress to episodes of SCAF >23 h as time progresses. While only around 30–40% of SCAF events are temporally related to stroke events, the presence of SCAF likely represents an important risk marker for stroke. Ongoing trials of anticoagulation in patients with SCAF durations less than 24 h will inform clinical practice and are highly anticipated. Further studies are needed to clarify the association between SCAF and clinical outcomes as well as the factors that modify this association.

## 1. Background

Subclinical atrial fibrillation (SCAF) is a term used to describe atrial fibrillation (AF) detected by cardiac devices in an asymptomatic patient [1]. These episodes are presumed to be of relatively short duration. A more accurate term is atrial high rate episodes (AHRE) given the difficulty in establishing that these episodes are indeed SCAF. AF is the one of the common arrhythmias encountered in clinical practice and is a major cause of preventable thromboembolic disease, namely stroke [2]. Early treatment of AF is considered essential to prevent stroke [3]; in up to 20% of AF cases, stroke may be the initial manifestation [4]. Whether SCAF is associated with stroke has been the subject of several studies [1] (references 16–19 should also be cited here).

Cardiac implantable electronic devices (CIED), namely pacemakers, implantable cardioverter defibrillators and implantable monitors are increasingly implanted worldwide. In 2009, 1.3 million CIEDs were implanted with over 400,000 CIEDs implanted in North America alone [5]. Specifically, there has been a significant increase in the implantation of pacemakers for sinus node dysfunction, which is associated with AF, as well as a significant absolute and relative increase in the use of dual-chambered pacemakers that provide an atrial lead which allows monitoring [6]. With an increased prevalence of CIEDs has come an increase in device detected AHRE (SCAF). This presents a conundrum to clinicians with regards to discussing the risk of clinical outcomes, namely stroke, with patients and whether these patients should be receiving oral anticoagulation. Does the duration of these episodes or burden of SCAF increase the risk of stroke? SCAF duration generally refers to the duration of a single episode of SCAF while SCAF burden refers to the daily burden of SCAF average over a certain period of time; the terms are sometimes used interchangeably.

Understanding the significance of SCAF is important with implications for clinical practice. This review assesses the risk of stroke associated with SCAF, the mechanisms underlying the association between SCAF and stroke as well as the effect of SCAF burden on stroke risk; finally ongoing trials that will inform clinical practice will be reviewed.

## 2. Importance of SCAF

Our initial understanding of the importance of SCAF stems from studies of patients who developed stroke. A significant proportion of strokes appeared to be of embolic origin with no clear cause after guideline-directed investigation for embolic causes [7]. This usually includes cardiac monitors for 24–48 h after the admission to screen for AF [8]. Despite extensive investigations, often no cause is found, and these patients are described to have an embolic stroke of undetermined source (ESUS) or cryptogenic stroke [7,9,10]. The Cryptogenic Stroke and Underlying Atrial Fibrillation (CRYSTAL AF) and the 30-Day Cardiac Event Monitor Belt for Recording Atrial Fibrillation after a Cerebral Ischemic Event (EMBRACE) trials have shown that with prolonged monitoring, including those via implantable cardiac devices, a significant proportion of patients with cryptogenic strokes have underlying asymptomatic AF episodes [11,12]. The importance of the detection of AF lies in its impact on subsequent management of stroke secondary prevention: Standard anti-platelet therapy if not related to AF and anticoagulation if due to AF [13]. Evidence from the EMBRACE and the CRYSTAL-AF emphasize the importance of undetected AF in terms of stroke risk [11,12]. While the majority (60%) of strokes are due to documented cerebrovascular disease, 15% are due to documented AF and 25% are due to ESUS [14]. Understanding the relationship between SCAF and stroke is thus imperative.

In the EMBRACE trial, patients were enrolled if they were 55 years or older, had experienced an ESUS event in the preceding six months which was confirmed by a stroke neurologist, and did not have AF and or another cause of stroke after extensive testing [12]. Patients were randomized in this open-label trial to undergo ambulatory electrocardiogram monitoring with a 30-day event-triggered loop recorder or one additional round of 24-h Holter monitoring [12]. Results of this trial showed AF lasting 30 s or longer was detected in 16.1% with the a 30-day event-triggered loop recorder compared to 3.2% in the control group (absolute difference, 12.9 percentage points; 95% confidence interval (CI), 8.0 to 17.6; *p* < 0.001; number needed to screen, eight) [12]. In the CRYSTAL-AF trial, investigators similarly enrolled patients who were 40 years and older who had experienced an ESUS event in the preceding three months that was supported by both symptoms and brain imaging and did not have a cause of ESUS after extensive testing [11]. Patients were randomized to receive either an implantable loop recorder (REVEAL XT) or conventional follow-up. At 12 months of follow-up, AF had been detected in 12.4% who received an implantable monitor compared to 2.0% of patients who received conventional therapy (hazard ratio (HR) 7.3; 95% CI, 2.6 to 20.8; *p* < 0.001) [11].

Two trials, New Approach Rivaroxaban Inhibition of Factor Xa in a Global Trial versus ASA to Prevent Embolism in Embolic Stroke of Undetermined Source (NAVIGATE-ESUS) and Randomized, Double-Blind, Evaluation in Secondary Stroke Prevention Comparing the Efficacy and Safety of the Oral Thrombin Inhibitor Dabigatran Etexilate versus Acetylsalicylic Acid in Patients with Embolic Stroke of Undetermined Source (RESPECT-ESUS), have evaluated a strategy of empiric anticoagulation for all patients with cryptogenic stroke [9,10]. These trials failed to demonstrate the superiority of oral anticoagulation over aspirin in the reduction of recurrent strokes in patients with ESUS [9,10]. This further highlights the importance of the detection of SCAF as well as understanding which patients with SCAF should receive treatment.

## 3. Stroke Risk

Several studies have assessed patients who had an implantable device for any indication and followed them for the development of AHRE [14,15,16,17,18,19]. These studies are summarized in Table 1. An implanted atrial lead allows for continuous detection and characterization of AHRE over a prolonged period of time [1]. Pollak and colleagues showed that a cut-off of five minutes for AHRE significantly reduces the risk that oversensing episodes are classified as SCAF [20].

In an ancillary study of the Mode Selection Trial (MOST) trial which randomized 2010 patients with sinus node dysfunction to dual chamber versus single chamber pacing, Glotzer et al. enrolled 312 patients and followed them for 27 months (median) with the atrial detection rate programmed to 220 beats per minute for at least 10 beats [16]. Analysis was limited to episodes of at least five minutes in keeping with the data by Pollack et al. [20]. Of the patients enrolled, 51% experienced at least one episode of AHRE (≥five minutes). In 160 patients with an AHRE, the primary endpoint of death or nonfatal stroke occurred in 33 patients (20.6%) compared to 10.5% in those without AHRE. AHRE was an independent predictor of death or stroke [16]. The Asymptomatic Atrial Fibrillation and Stroke Evaluation in Pacemaker Patients and the Atrial Fibrillation Reduction Atrial Pacing Trial (ASSERT) trial enrolled 2580 patients who were ≥65 years old, had hypertension but no history of AF who had received a CIED (from St Jude Medical) for sinus node or atrioventricular node dysfunction in the eight weeks prior to enrollment [1]. Patients were excluded if they had any history of AF or atrial flutter or if they required oral anticoagulation for any indication. These patients were then monitored for three months to assign patients into two groups, those with AHRE (defined as an atrial rate of 190 beats or more lasting more than six minutes) and those without AHRE. The patients were subsequently followed every six months for a mean of 30 months for the primary outcome which consisted of systemic embolism or stroke [1]. At the three-month monitoring period, SCAF had occurred in 10.1% of patients. SCAF was associated with a five-fold increased risk of clinical AF (HR 5.56; 95% CI 3.78 to 8.17; *p* < 0.001) and a 2.5-fold increased risk of stroke or systemic embolism (HR 2.49; 95% CI, 1.28 to 4.85; *p* = 0.007). After adjustment for stroke predictors, SCAF remained predictive of the stroke or systemic embolism [1]. Though the stroke risk is not as high as that seen with clinical AF, which is four to five times the general population, it is still significant at two to two-and-a-half times the general population [22]. The major limitation of the ASSERT trial is that SCAF was defined using a limited sampling period of three months post device implantation. SCAF, in that time-period may have been transient due to lead implantation (Mittal et al. 2008). These trials also do not differentiate the type of strokes due to limitations with data. The data from MOST and ASSERT clearly demonstrate that patients with SCAF have an associated risk of stroke but a risk lower than clinical AF. Further studies and analyses are required to better improve our understanding of SCAF

An important observation of the above-mentioned studies is that SCAF was detected in patients with implantable pacemaker and defibrillators who were therefore at higher risk of developing AF. Whether this risk translates into increased risk in the general population were they to be monitored remained controversial. Several studies have attempted to address this issue by having cardiac monitors implanted for the purpose of monitoring for SCAF. These studies are also included in Table 1. In an international prospective, single-arm, multicenter study (REVEAL-AF), conducted from November 2012 to January 2017, 386 patients with at least three risk factors for stroke were enrolled after initial screening [23]. Participants received an implanted cardiac monitor for a mean of 22.5 months (Reveal XT or Reveal LINQ; Medtronic). The primary end point was adjudicated AF lasting six or more minutes and was assessed at 18 months. In addition, the median time from device insertion to SCAF detection was also assessed as was the subsequent prescription rate for anticoagulation. The incidence rate of significant SCAF ≥ six minutes progressively increased with longer monitoring: 6% at 30 days, 20% at six months, 27% at one year, 34% at two years and finally 40% at 30 months. The median time from insertion to detection of SCAF was three months and 72 patients (56% of those who developed SCAF at the 18-month primary end-point of the trial) received oral anticoagulation [23]. Similarly, ASSERT-II was a prospective single-arm multi-center study that enrolled 256 patients from cardiology and neurology clinics who had no history of AF and at least two risk factors for stroke [21]. In addition, patients were required to have an element of cardiomyopathy manifest as either an enlarged left atrium or elevated brain natriuretic peptides. Significant SCAF was defined as episodes lasting longer than five minutes and follow-up was for 17 months. The incidence of SCAF was 34% which was predicted by age, hypertension and an enlarged left atrium [21]. The major knowledge gap in this area is patient selection given the lack of clinical outcomes and cost-effectiveness analyses.

## 4. SCAF Burden

Whilst SCAF was clearly associated with an increased risk of stroke, uncertainty remained as to whether longer episodes were more likely to be associated with stroke. In a scientific statement, the American Heart Association acknowledges the large knowledge gaps pertaining to AF burden. In particular, the statement notes the burden at which clinical outcomes increase remains unknown [24]. TRENDS (A Prospective Study of the Clinical Significance of Atrial Arrhythmias Detected by Implanted Device Diagnostics) was a prospective observational study in patients with a CIED and at least one risk factor for stroke [15]. Patients were excluded if they had long-standing persistent AF, re-entrant supraventricular arrhythmias or a terminal illness. Interestingly, patients with paroxysmal AF were included in this study. Follow-up visits occurred every three months, but the study was terminated early due to a low event rate. AHRE detection settings were set at an atrial rate of greater than 175 beats per minute lasting greater than 20 s. The authors concede that these settings would not help differentiate AF from other atrial tachyarrhythmias [15]. In addition, with such a short detection duration, the device was liable to record over-sensed events as AHRE. Patients were classified into three groups based on the longest duration of AHRE during 30-day window subsets: Zero burden, low burden (≤5.5 h) and high burden (>5.5 h). During a mean follow-up of 1.4 years, the annualized thromboembolic (stroke and transient ischemic (TIA)) risk was 1.1% in the zero-burden group, 1.1% in the low burden group, and 2.4% in the high burden group. In comparison to those with zero-burden, the adjusted HR in the low and high burden subsets were 0.98 (95% CI 0.34 to 2.82, *p* = 0.97) and 2.20 (95% CI 0.96 to 5.05, *p* = 0.06), respectively [15]. The inclusion of patients with history of paroxysmal AF was a major limitation of this study. In a systematic review and meta-analysis, Mahajan et al. pooled data from seven studies. The duration cut-off for AHRE varied among the included studies. In patients with subclinical AF exceeding the defined cut-off SCAF duration of the study, the annual stroke rate was 1.89/100 person-year with a 2.4-fold (95% CI 1.8–3.3, *p* < 0.001) increased risk of stroke compared to patients with subclinical AF who did not reach the cut-off duration; the absolute risk was 0.93/100 person-years [25].

Attempts were made to assess whether increased AF burden was associated with an increased risk of stroke. Proietti and colleagues performed a systematic review and meta-analysis to assess whether SCAF burden is associated with stroke risk [26]. This analysis was limited by a small number of studies that reported such data and the varying cut-off points used in the studies. The authors concluded that a direct correlation between burden of asymptomatic AF and HR for stroke cannot be confirmed [26]. Van Gelder and colleagues performed an important analysis of the ASSERT study which showed that SCAF for a duration >24 h is associated with an increased risk of ischemic stroke or systemic embolism [27]. Patients were divided into groups depending on the duration of the single longest duration of SCAF: 19% > six minutes to 6 h, 7% > six hours to 24 h and 10.7% > 24 h; patients with SCAF for <6 min were excluded from this analysis; patients were followed for 2.5 years. In patients in whom the longest episode of SCAF exceeded 24 h, there was an associated significant increased risk of stroke (adjusted HR 3.24, 95% CI 1.51–6.95, *p* = 0.003). In patients with SCAF between six minutes and 24 h, the risk of stroke was not significantly different from patients without SCAF [27]. Patients who had SCAF for ≥24 h had an annual stroke risk of approximately 5% which is similar to the risk observed in patients with clinical AF. The significant increase in stroke risk with episodes > 24 h was also noted in the AT500 registry [18]. These studies have informed the current practice of prescribing oral anticoagulation in patients with SCAF episodes > 24 h but questions remained regarding patients with long episodes of SCAF not reaching 24 h and those who experience progression to longer SCAF episodes. Going forward, another metric, such as AF density may become clinically relevant. AF density incorporates the temporal dispersion of AF burden (Charitos et al.); further studies are needed to compare the effects of AF burden and AF density.

## 5. Progression

Whether progression to longer episodes of SCAF is associated with an increased risk of stroke is unclear. Initial data came from anticoagulation studies that compared the risk of stroke in patients with paroxysmal compared to more persistent forms of AF. In the Apixaban for Reduction in Stroke and Other Thromboembolic Events in Atrial Fibrillation (ARISTOTLE) trial, in which patients with AF were randomized to apixaban versus warfarin, the incidence of stroke or systemic embolism was significantly higher in patients with persistent or permanent AF compared to patients with paroxysmal AF (1.52 vs. 0.98%; *p* = 0.003, adjusted *p* = 0.015) [28]. In the Rivaroxaban Once Daily Oral Direct Factor Xa Inhibition Compared with Vitamin K Antagonism for Prevention of Stroke and Embolism Trial in Atrial Fibrillation (ROCKET-AF) trial, patients with AF were randomized to receive either rivaroxaban or warfarin. There was a significantly higher risk or stroke (2.18 vs. 1.73 events per 100-patient-years, *p* = 0.048) and death (4.78 vs. 3.52 events per 100-patient years, *p* = 0.006) in patients with persistent AF compared to paroxysmal AF [29]. Data from clinical AF suggests that progression to a greater AF burden increases stroke risk. De Vos showed that approximately 15% of patients with paroxysmal AF progress to persistent AF at one year of follow-up [30]; this progression is predictable using clinical risk scores such as the Hypertension, Age, Transient ischaemic attack or stroke, Chronic obstructive pulmonary disease, and Heart failure (HATCH) score [30,31] and may be preventable with catheter ablation [32]. Patients who progress are more likely to become symptomatic or experience a stroke or TIA [30].

**Figure 1:**
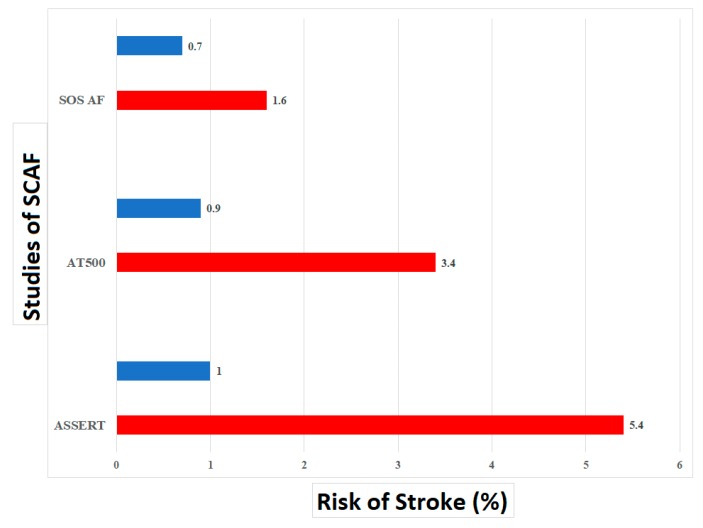
The risk of stroke associated with a SCAF burden ≥23 h in different studies compared to no SCAF. Risk of stroke in patients with SCAF ≥23 h (red) compared to no SCAF (blue). SCAF = subclinical atrial fibrillation.

Similar findings were found in patients with SCAF. Boriani et al. pooled patient level data from three prospective studies: TRENDS, Stroke preventiOn Strategies based on Atrial Fibrillation information from implanted devices (SOS) and Phase IV Long Term Observational Study of Patients Implanted With Medtronic CRDM Implantable Cardiac Devices (PANORAMA) [33]. Among the study population of 6580 patients, de novo AF with a SCAF burden of ≥5 min, was detected in 2244 patients (34%) during a follow-up period of 2.4 ± 1.7 years. Among these patients, 1091 (49.8%) transitioned to a higher SCAF-burden threshold during follow-up. Approximately 24% of patients transitioned from a lower threshold to a daily SCAF burden of ≥23 h during follow-up. Factors associated with transition to a greater SCAF burden on multivariate analysis included male gender and a CHADS2 (Congestive heart failure, Hypertension, Age>75, Diabetes mellitus and Stroke or transient ischemic attack) score of two or greater (33)]. Figure 1 shows the risk associated with a SCAF burden ≥ 23 h in various studies compared to no SCAF. Wong et al. also performed a sub study of ASSERT to assess the impact of SCAF burden progression. Patients in whom the longest SCAF episode was >6 min but <24 h during the first year (415 patients) were included [34]. The authors assessed the association between progression to SCAF >24 h or the development of clinical AF and heart failure hospitalizations. During a mean follow-up of two years, 15.7% of patients progressed. The rate of heart failure hospitalization among patients with SCAF progression was 8.9% per year compared with 2.5% per year for those without progression. After multivariable adjustment, SCAF progression was independently associated with HF hospitalization (HR 4.58; 95%; CI: 1.64 to 12.80; *p* = 0.004). These results remained significant even if patients with a history of heart failure were excluded or when the analysis was limited to only progression to SCAF >24 h and not clinical AF [34]. Therefore, it seems that a significant SCAF burden of greater >24 h is associated not only with an increased risk of stroke but also an increased risk of heart failure hospitalizations [22]. This is consistent with the relationship seen between clinical AF and heart failure in which a vicious cycle can develop [35].

## 6. Mechanism of Increased Stroke in SCAF

**Figure 2:**
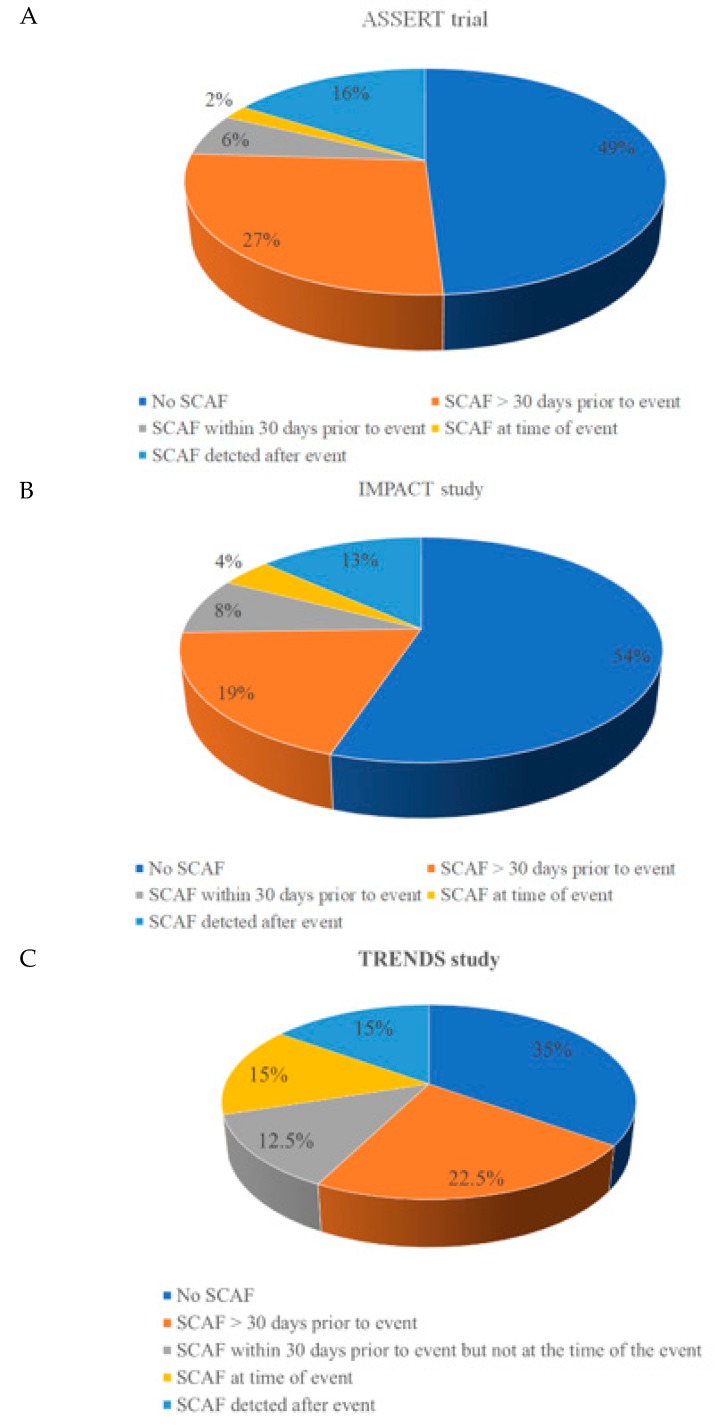
SCAF and stroke: A temporal relationship based on data from (**A**) ASSERT, (**B**) IMPACT and (**C**) TRENDS. All stroke or systemic embolism events are correlated with SCAF.

A direct and causal relationship between SCAF and stroke is not entirely clear. In its scientific statement the American Heart Association recognizes the presence of an important knowledge gap with regard to whether a temporal relationship exists between AF burden and stroke risk [24]. The association between AF and stroke is complex. There is a strong causal association between AF and embolic stroke but also an association with ischemic stroke [36]. In addition to causing atrial thromboembolism through stasis and clot formation, other factors such endothelial dysfunction, left atrial fibrosis myocyte dysfunction, chamber dilatation and left atrial appendage mechanical dysfunction may also have a role in stroke risk [37]. Brambatti and colleagues performed a sub-analysis of the ASSERT trial to assess the temporal association between SCAF and stroke and systemic embolism; the analysis included SCAF >6 min [38]. Of all the patients who experienced a stroke or systemic embolism, 51% had SCAF in keeping with previous data that shows that 50–60% of strokes are due to atherosclerosis and not thromboembolism. However, of the patients experiencing SCAF, only 16% had SCAF in the 30 days preceding the stroke or systemic embolism event [38]. A sub-analysis of the TRENDS study had similar findings; only 11 (27.5%) of the 40 patients developing clinical thromboembolism exhibited SCAF within 30 days before the event [39]. Figure 2 depicts the temporal relationship between SCAF and stroke in three studies [40].

There are several important points to note from this data in the context of our current understanding of AF. For the majority of stroke or systemic embolism events, there was no temporal relationship with SCAF. When a temporal relationship existed, the duration of SCAF was almost always much shorter in duration than the 48 h commonly believed to be the minimum duration required for thrombus to form in the left atrial appendage; this is the time period considered safe for cardioversion [41]. Furthermore, those with SCAF who experienced stroke did not require progression to longer episodes in order to develop stroke or systemic embolism. Therefore, SCAF is likely an important risk marker for stroke and systemic embolism and if there is a direct causal relationship this is not as simple as a direct predictable relationship.

## 7. Treatment

**Figure 3:**
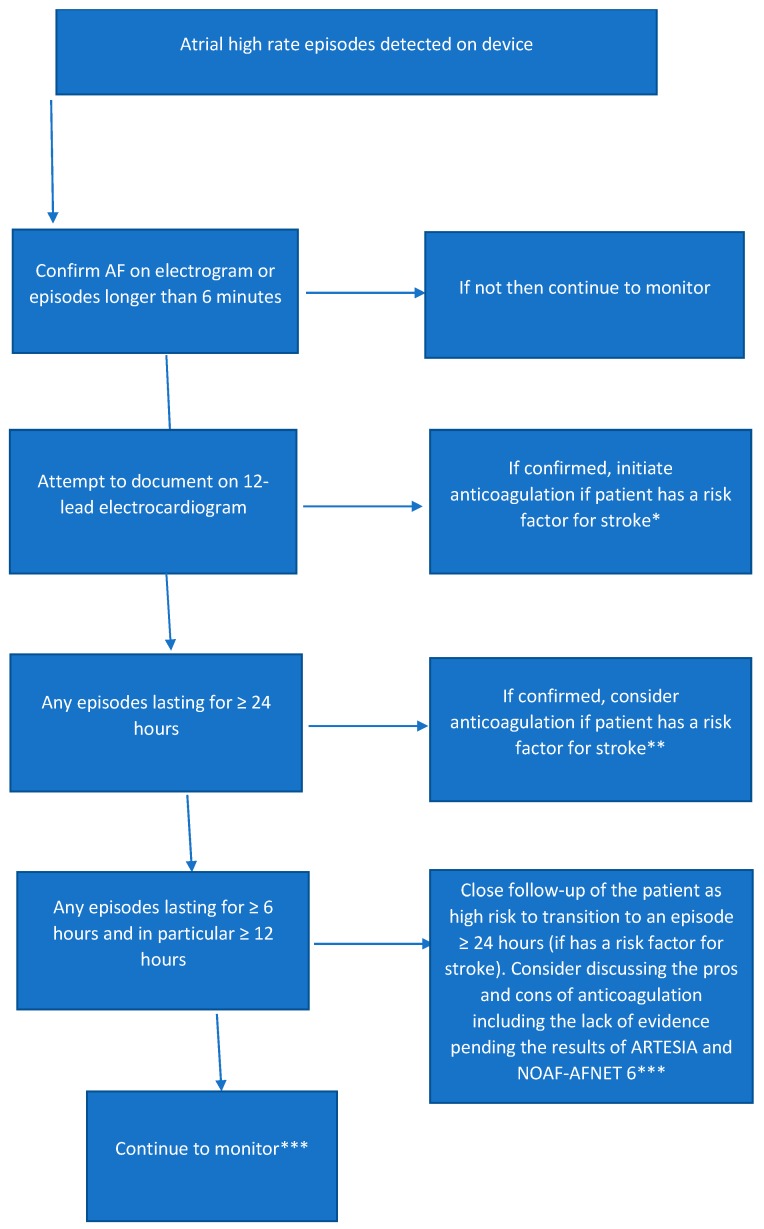
Suggested clinical algorithm for the management of SCAF. * Based on current atrial fibrillation guidelines [2,42]; ** based on current guidelines to consider anticoagulation when a device detected episode is for 24 h or longer [42]; *** data suggests that these patients are at high risk to transition to episodes lasting 24 h or longer [33]. *** Management for patients with an episode > 6 min is currently unclear and such patients may be eligible for enrolment in the ARTESIA and NOAH-AFNET 6 trials depending on the presence of other risk factors for stroke.

The treatment of SCAF presents several challenges due to the issues raised above. There are several factors to keep in mind when considering the treatment of SCAF. In clinical AF, oral anticoagulation is recommended regardless of AF subtype and depending on the presence of clinical factors (age, hypertension, diabetes mellitus, heart failure and stroke) which have consistently been shown to increase stroke risk [42]. Given that oral anticoagulation has been shown to significantly decrease stroke risk in clinical AF, this risk reduction should theoretically translate to SCAF. However, in SCAF the increased risk is of a lower magnitude (2–2.5 times) compared to clinical AF (5 times) and this may reduce the net clinical benefit observed with anticoagulation in SCAF. Patients with SCAF >24 h have an absolute risk profile that is similar to that observed in clinical AF and are the subgroup of SCAF most likely to derive benefit from oral anticoagulation. This is consistent with current guidelines [42]. An algorithm for the management of SCAF is proposed in Figure 3.

The equipoise in whether patients with SCAF should receive oral anticoagulation can be observed in clinical practice. Healey et al. performed a retrospective analysis of all patients at a single academic hospital who had pacemakers capable of documenting AF [43]. In 445 patients studied, SCAF was found in 55% of patients who were more likely to be older, have history of clinical AF and a large left atrium. Anticoagulants were used more frequently among patients who also had clinical AF (58.9%) compared with those without (23.7%, *p* < 0.001) [43].

One strategy that was attempted is intermittent anticoagulation during episodes of SCAF with the premise that stroke risk is highest at that time-point while avoiding the risk of bleeding at other times. In the Multicenter Randomized Trial of Anticoagulation Guided by Remote Rhythm Monitoring in Patients with Implanted Cardioverter-Defibrillator and Resynchronization Devices (IMPACT) trial, 2718 patients with CIEDs were randomized to start and stop anticoagulation based on remote rhythm monitoring compared to usual office-based follow-up with anticoagulation determined by standard clinical criteria [44]. The primary endpoint was a composite of stroke, systemic embolism, and major bleeding. The trial was stopped early after a two-year median follow-up due to futility. Primary events (2.4 vs. 2.3 per 100 patient-years) were similar between the two groups (HR 1.06; 95% CI 0.75–1.51; *p* = 0.732) [44]. There are multiple issues that limit inferences from this trial. Firstly, it was performed in the era of warfarin which limits the ability to provide rapid intermittent anticoagulation and which increases the risk of bleeding. In addition, because major bleeding was used in the primary endpoint composite, this may have led to the neutral result (observed and early termination. In addition, the algorithm for home monitoring was complex with poor adherence. In the end, anticoagulation use was similar in both arms [44].

## 8. Future Directions

Current guidelines suggest that patients with AHRE (SCAF) greater than 24 h as well as at least one risk factor for stroke should receive oral anticoagulation [42]. In addition, the guidelines also suggest that patients with shorter durations of AHRE who are at high risk such as those with cryptogenic stroke should be considered for oral anticoagulation [42]. The gap in knowledge exists for patients who have episodes between 6 min and 24 h, particularly in patients with relatively longer episodes. Given the clinical equipoise in this population, two trials are currently underway to try and provide clarity: The Apixaban for the Reduction of Thrombo-Embolism in Patients With Device-Detected Sub-Clinical Atrial Fibrillation (ARTESIA) trial and the Non-vitamin K Antagonist Oral Anticoagulants in Patients With Atrial High Rate Episodes (NOAH-AFNET 6) trial [45,46].

ARTESIA is a prospective, multicenter, double-blind, randomized controlled trial, enrolling patients with SCAF detected by a CIED who have additional risk factors for stroke [46]. To be eligible, participants must have had at least one episode of SCAF ≥6 min in duration on device interrogation, be at least 55 years of age or older and have risk factors for stroke (the number of risk factors depends on the age with those 75 years of age or older not requiring any further risk factors) [46]. The trial will exclude patients with documented AF on a 12-lead electrocardiogram or those who have an indication for oral anticoagulant therapy. Participants will be randomized to apixaban or aspirin 81 mg daily and will receive placebo pills accordingly (aspirin and two placebo pills instead of apixaban or apixaban and placebo aspirin) [46]. The primary outcome is the composite of stroke, TIA (with magnetic resonance imaging evidence of a cerebral infarction on diffusion-weighting) and systemic embolism. The trial is aiming to recruit around 4000 patients from 230 international clinical sites. ARTESIA is expected to have 36 months of follow-up until 248 adjudicated primary outcome events have occurred [46].

NOAH-AFNET 6 is an investigator-driven, prospective, parallel-group, randomized, event-driven, double-blind, multicenter trial [45]. The trial will recruit patients with SCAF detected by a CIED with at least one risk factor for stroke. To be eligible, participants must have AHRE, be aged ≥ 65 years with at least one other stroke risk factor. Excluded will be patients with documented AF or an indication for oral anticoagulation. These broad inclusion/exclusion criteria were put in place to mimic clinical practice. NOAH-AFNET 6 will randomize 3400 patients to edoxaban or no anticoagulation (aspirin depending on clinical indications) in a superiority trial. The primary efficacy outcome is stroke or cardiovascular death and the primary safety outcome will be major bleeding. All patients will be followed until the 222 target primary outcomes are reached. Patients will be censored when they develop AF and offered open-label anticoagulation [45].

Further studies are needed to improve our understanding of SCAF, its clinical impact and the role of different therapies. A prospective study of well-matched patients with SCAF compared to clinical AF would provide insight into the true incidence of thromboembolic events in both groups. In addition, a greater understanding of the temporal relationship between SCAF and thromboembolic events is needed which would need a larger study. Does atrial AF burden or density significantly increase risk; and consequently, does the reduction of SCAF burden mitigate this risk? Studies in clinical AF suggest that this is unlikely to be the case. Finally, does anticoagulation therapy in patients with high SCAF burden/density, those who have significant progression of SCAF or patients with evidence of an underlying cardiomyopathy (enlarged left atrium or elevated natriuretic peptides) significantly reduce the risk of thromboembolic events?

There are several noteworthy limitations and unanswered questions regarding SCAF with current data. Not all stroke events in SCAF studies are embolic strokes and as shown above with the timing of stroke relative to the SCAF episode, SCAF may be a risk marker for stroke. Another important element to consider when discussing AF and SCAF burden is the large discrepancy between clinical and device detected categorization of the pattern of AF. Charitos and colleagues showed that while the majority of patients with AF are clinically classified as paroxysmal AF, this correlates poorly with the temporal persistence of AF based on continuous device monitoring [47]. The impact of this discrepancy on clinical practice remains unclear. Finally, the highly anticipated results of the two anticoagulation in SCAF trials should be released in 2022 with the large potential to change clinical practice.

## 9. Conclusions

Cardiac implantable electronic devices have led to the detection of SCAF which has clearly been shown to be associated with an increased risk of stroke. Based on current data, patients with episodes of SCAF lasting for 24 h or greater and at least one risk factor for stroke should receive oral anticoagulation. Longer episodes of SCAF and progression to longer episodes also confer an increased risk of stroke. Trials are now underway to assess the efficacy and safety or oral anticoagulation in patients with SCAF lasting less than 24 h.

## Figures and Tables

**Table 1:** The incidence of subclinical atrial fibrillation (SCAF) and associated stroke in patients with cardiac implantable electronic devices (CIEDs).

Study (First Author, Year)	Study Population	Design	Mode of SCAF Detection	SCAF Criteria + Burden	Clinical Outcome	Annual Stroke and Systemic Embolism Event Rate (%)	Comparative Outcomes (HR, 95% CI)	F/u	Incidence of SCAF (%)
**ASSERT (Healey et al., 2013)** [1]	2580 patients Inclusion criteria: ≥65 years HTN requiring medical therapy Initial implantation of a St. Jude Medical dual-chamber pacemaker (for sinus-node or atrioventricular-node disease) or defibrillator (for any indication) in the preceding 8 weeks. Exclusions: History of atrial fibrillation or flutter or an indication for oral anticoagulation	Prospective cohort study	Dual chamber pacemaker or defibrillator	>190 bpm + ≥6 min	Stroke or systemic embolism	SCAF = 1.69 No SCAF = 0.69	5.56, 1.28–4.85	2.5 years	10.1
**ASSERT II (Healey et al., 2017)** [21]	256 patients Inclusion criteria: ≥65 years AND CHA2DS2-VASc score > 2, OR obstructive sleep apnea, OR BMI > 30, AND Left atrial enlargement OR elevated serum N-terminal pro–brain-type natriuretic peptide level ≥ 290 pg/mL	Prospective cohort study	CONFIRM AF subcutaneous cardiac monitor	+ ≥6 min	NA	6 events occurred (4 stroke, 1 TIA and 1 systemic embolism) but none in patients who had SCAF detected	NA	16 months	34.4
**AT 500 registry (Capucci et al., 2005)** [18]	225 patients Inclusion criteria: Bradycardia Guideline indication for dual-chamber pacing History of documented symptomatic atrial tachyarrhythmias	Prospective cohort study	Dual chamber pacemaker	NA + 24 h	Stroke or systemic embolism	NA	3.10, 1.10–10.50	22 months	NA
**MOST (Glotzer et al., 2003)** [16]	312 patients Inclusion criteria: ≥21 years Dual-chamber pacemaker for sinus node dysfunction In sinus rhythm at onset of study	Prospective cohort study	Dual chamber pacemaker	>220 bpm + ≥5 min	Stroke and all-cause mortality	SCAF = 1.82 No SCAF = 0.48	2.79, 1.51–5.15	33 months	51.3
**HOME CARE and EVEREST (Shanmugam et al., 2011)** [19]	560 patients All patients had heart failure and a biventricular pacemaker capable of continuous heart rhythm monitoring though home monitoring. Patients in sinus rhythm (including patients with a prior history of AF) with >70% home monitored transmissions during follow up (minimum >3 months follow-up)	Prospective cohort study	Biventricular pacemaker or defibrillator	>180 bpm + ≥3.8 h	Stroke or systemic embolism	NA	9.40, 1.80–47.00	12 months	40
**SOS-AF (Boriani et al., 2014)** [17]	10,016 patients Inclusion: CIED able to monitor for atrial high rate episodes At least 3 months of follow-up Device diagnostic data available Did not have permanent AF	Prospective cohort study	CIED	>175 bpm + ≥5 min	Stroke	SCAF = 0.49 No SCAF = 0.31	1.76, 1.02–3.02	24 months	43
**TRENDS (Glotzer et al., 2009)** [15]	2486 patients Inclusion criteria: Guideline indication for an implantable cardiac rhythm device capable of long-term monitoring At least 1 risk factor for stroke Exclusion criteria: Replacement devices : Long-standing persistent AF Known re-entrant supraventricular tachycardias, Terminal illness limiting survival Unable or unwilling to consent Enrolled in a conflicting drug or device study.	Prospective cohort study	CIED	>175 bpm + ≥5.6 h	Stroke or systemic embolism	SCAF = 2.4 No SCAF = 1.1	2.20, 0.96–5.05	1.4 years	55.9

SCAF = Subclinical atrial fibrillation; HR = hazard ratio; CI = confidence interval; FU = follow up; CIED = cardiac implantable electronic device; TIA = transient ischemic attack; NA = not available; HTN = hypertension; BMI= body mass index; CHA2DS2-VASc = congestive heart failure, hypertension age, diabetes mellitus, stroke, vascular disease, age and sex.
